# Uncertainty of Artificial Intelligence Assistant: The Effect of Assistant Type on Variety Seeking

**DOI:** 10.3389/fpsyg.2022.904302

**Published:** 2022-05-26

**Authors:** Yu Zhang, Mengya Yang, Ziling Zhang

**Affiliations:** ^1^School of Management, Tianjin University of Technology, Tianjin, China; ^2^Business School, Nankai University, Tianjin, China; ^3^Alliance Manchester Business School, The University of Manchester, Manchester, United Kingdom

**Keywords:** artificial intelligence, assistant type, uncertainty, involvement, variety seeking

## Abstract

In service marketing, AI assistants and self-service technology have become popular. As a result, it is critical to enrich the understanding of whether consumers react differently in the artificial intelligence (AI) service context in comparison with the human service context. This study examines the effect of assistant type (AI vs. human) on consumers’ decision-making. Through three experiments, this research finds that variety seeking will be higher when consumers are making decision in AI (vs. human) service environment. Furthermore, we tested uncertainty as the underlying mechanism. Moreover, we demonstrated that this pattern is moderated by situational involvement. Specifically, in consumption contexts of high involvement, the consumers are less likely to seek variety, and in consumption contexts of low involvement, they prefer more variety (study 3). This research offers service providers new insights by revealing how, why, and when the interaction of AI technology influences consumers’ decision-making in service marketing.

## Introduction

Artificial intelligence (AI) is a powerful and sophisticated technology aimed at simulating human intelligence (HI), which is at the heart of the “Fourth Industrial Revolution,” in which the lines between the physical, biological, and digital realms are becoming increasingly blurred (Schwab, 2017). In the service industry, AI is rapidly being used and becomes a notable source of innovation, which is characterized by the shift of agency and control from human being to technology, and thus alters our past view of human-technology relationship (Rust and Huang, 2014).

The existing service literature is mostly concerned with the use of intelligent technology (Marinova et al., 2017; Rafaeli et al., 2017), service technologies (Kunz et al., 2018), and service greatly aided by a wide range of technologies (Huang and Rust, 2013). According to studies, technology advances should have foreseeable implications, including increased optimal productivity (Rust and Huang, 2012), an increase in the usage of self-service technologies (Meuter et al., 2000), and a better-developed service sector (Rust and Huang, 2014). As AI is being increasingly widely used in the service frontline, how the AI service context influences consumers’ decision-making is a research question worthy of attention.

In this study, we performed beyond technology to understand how psychological mechanisms impact consumers’ variety-seeking behaviors in the context of AI services. The need for variety is an important aspect of decision-making. We eat salad for lunch since we ate a sandwich the day before, or we buy a variety of chips rather than many bags of the same flavor. Consumers need variety (Kahn, 1995), whether it is in the form of a single item to consume now or a portfolio of possibilities to enjoy later (Simonson, 1990). We propose that consumers will increasingly seek variety when making decision in AI service environment than when they do in human service environment. Due to the complexity and potential non-determinism of AI behaviors, uncertainty embeds in human-AI relationships (Menosky, 2017).

As a result, we hypothesized that this effect happens as a result of the uncertainty consumers experience when interacting with AI robots, and the increased variety helps to alleviate this discomfort (Levav and Zhu, 2009). We further believe that the impact is enhanced when customers purchase low- (vs. high-) involvement products because they tend to process information bypassing the peripheral route.

By documenting the novel effect of AI service context on consumer variety-seeking behaviors, this research links the studies on the application of AI technology in the service sector and consumer choice and reveals consumers’ psychological decision-making mechanism in AI service context, laying the groundwork for future study in this growing area. We established the theoretical framework, develop our hypotheses, provided our three experimental studies, and concluded with a discussion of our contributions, suggestions for future research, and managerial implications in the following sections.

## Conceptual Framework and Hypotheses

### Artificial Intelligence in Service Context

Artificial intelligence, which is defined as machines that display human intelligence (HI), becomes a key source of innovation and is increasingly being used in the service industry (Rust and Huang, 2014). Robots working for homes, hotels, restaurants, supermarkets, and healthcare, for example, have automated our lives in a variety of ways—AI applications powered by big data are taking the place of portfolio managers (Javelosa, 2017); virtual assistants or bots have changed consumer service into self-service (Fluss, 2017); and social robots like Pepper are being used in frontline services to replace human greeters (Choudhury, 2016). Robotics are expected to progressively replace human workers, even in complicated, analytical, intuitive, and empathetic activities (Huang and Rust, 2018).

Customers’ psychological reactions to robot aesthetics (e.g., human-likeness), which may impact consumers’ comfort during service robot encounters, have been studied in a few recent small-scale empirical works (Mende et al., 2019; Van Pinxteren et al., 2019). For instance, in the context of an AI service, more human-likeness of the robots may boost consumer discomfort, prompting them to exhibit compensatory behaviors (e.g., purchasing status goods, seeking social affiliation, and eating more; Mende et al., 2019). Despite this, there are few marketing studies on service robots (Huang and Rust, 2018; Wirtz et al., 2018).

### Drivers Underlying Variety Seeking

Studies have been undertaken over decades to determine the effect of variety seeking on consumer behaviors (Kahn, 1995). From everyday decisions such as what to eat to major ones such as what to do with one’s time, consumers seek out and are impacted by the variety on a regular basis (Kahn, 1995; Broniarczyk et al., 1998; Redden and Hoch, 2009). Variety seeking is so established in humans that for the purpose of variety, individuals sometimes even choose less-favored products (Ratner et al., 1999; Ariely and Levav, 2000).

Theoretical arguments and boundary conditions for consumer variety seeking are provided by previous studies. First, the motivation to reduce satiation may induce variety seeking (McAlister, 1982; Sevilla et al., 2019). Second, studies indicate variety seeking as a way of reducing the extent of uncertainty regarding future desires and obtaining information (Kahn and Lehmann, 1991; Ariely and Levav, 2000). Third, people desire variety in their decision-making so that their retrospective experiences are enhanced, along with their overall satisfaction with their experiences (Ratner et al., 1999). Apart from these, variety seeking also helps to avert future regret (Ariely and Levav, 2000). Fourth, self-presentation motivation is also a driver of variety seeking (Ratner and Kahn, 2002). For example, due to the incentive of self-presentation, variety seeking was stronger in the public context than in the private consumption. Fifth, variety seeking may be used to increase one’s sense of control and freedom (Levav and Zhu, 2009; Yoon and Kim, 2018). When options were physically constrained, such as purchasing in a small (vs. large) aisle, Levav and Zhu (2009) reported significantly higher levels of variety seeking. Furthermore, consumers who assessed their economic mobility to be low (vs. high) exhibited significant variety-seeking behavior (Yoon and Kim, 2018). Finally, under simultaneous-choice (vs. separate-choice) settings, variety seeking is higher (Simonson, 1990; Simonson and Winer, 1992; Read and Loewenstein, 1995). Specifically, it is demonstrated that the number of choices made at one time had an effect on the choice variation, with consumers in the simultaneous-choice condition being more likely to pick a variety of items than those in the sequential-choice condition. As a result, earlier research reveals that variety seeking is a common phenomenon among consumers and in daily life. In this study, we focused on the impact of AI service as a situational factor on variety seeking.

### The Relationship Between Artificial Intelligence Service Environment and Variety-Seeking Behavior: The Mediating Role of Uncertainty

Artificial intelligence has made its way into the marketing world and is having a significant influence on consumer decision-making. In the context of consumer services, AI is a technology-enabled system that evaluates real-time service scenarios using data collected from digital and/or physical sources to provide personalized recommendations, alternatives, and solutions to consumers’ inquiries or problems, even the most complex ones (Xu et al., 2020). Thus, when AI is incorporated into a consuming scenario, it becomes a unique situational factor, which could possibly influence consumer behaviors like other situational factors such as time of the day, place, temperature, and color.

Despite its various advantages (e.g., in medical imaging; Pesapane et al., 2018), AI service environment can also be threatening for consumers, leading to the feeling of uncertainty. From four decades ago, researchers have noticed consumers’ perceived technology threats and named it technophobia (Naiman, 1982; Kassner, 1988). As the technological landscape grows and becomes more pervasive, many users’ anxieties about losing control and privacy have grown even to clinical proportions. Scales like the Cyber-Paranoia and Fear Scale (Mason et al., 2014) and the Food Technology Neophobia Scale (Cox and Evans, 2008) were developed to measure an individual’s technology-related fears and threats. AI, employed in a variety of technologies and even portrayed as a thinking machine, is also considered a threat. For instance, the ability of retailers like Amazon Go to follow every shopper’s movement using cameras, machine learning, and computer vision, for example, is criticized for undermining consumer privacy (Menosky, 2017). Stephen Hawking even had an extreme position, believing that a thinking machine may endanger humanity’s existence. Yogeeswaran et al. (2016) observed that highly humanlike robots can be seen as a realistic danger to human career paths, resources, and safety, as well as a threat to human identity and distinctiveness, particularly if those robots also perform better than humans.

In sum, the core of the preceding discussion of concerns about technologies in general, and AI-enabled technologies in particular, is that consumers may regard the evolving technological environment as riddled with uncertainty, which is ubiquitous in real-life situations and creates a significant barrier to decision-making.

We expect that AI service environment and the uncertainty it induced will positively influence variety seeking. Higher variety seeking in AI service environment may be motivated by the underlying mechanism of eliminating uncertainty and enhancing one’s personal control. When consumers interact with unfamiliar AI facilitates, their feelings of control are constrained, resulting in a strong need to restore control. This prediction is supported by Brehm’s reactance theory (Brehm, 1966; Brehm and Brehm, 2013). According to reactance theory, individuals who have a restricted feeling of control generate a high motivational state aimed at restoring control that has been threatened or taken away. As a result, one of the most common outcomes of psychological reactance is an individual’s attempt to restore control through behavior. In addition, a desire for more diversity is closely connected to restoring one’s sense of control (Levav and Zhu, 2009; Yoon and Kim, 2018). As a result, reactance theory argues that when people’s uncertainty grows and their sense of control diminishes, they would prefer a high level of variety. Therefore, we expect when consumers are purchasing in an AI service environment, and due to the uncertainty they feel, they are more prone to seek variety in their product choosing behaviors. Formally:

H1: Variety seeking will be higher (vs. lower) when consumers are making decision in AI (vs. human) service environment.

H2: Sense of uncertainty mediates the relationship between AI (vs. human) service environment and consumers’ variety-seeking behaviors.

### The Moderating Role of Situational Involvement

Involvement is defined as an internal state that indicates the amount of arousal, interest, or drive elicited by a product class (Dholakia, 2001). Consumer studies distinguish between two distinct forms of consumer involvement: enduring involvement, a variable that measures individual differences, represents consumer’s persistent interest in a product category (Bloch and Richins, 1983; Dholakia, 2001), and situational involvement, a state in which the consumption issue becomes more important, and in order to make better decisions, the consumer allocates more cognitive resources (Houston and Rothschild, 1978; Roser, 1990).

In the service context, we believe that the level of situational involvement should moderate the influence of AI service environment on a consumer’s variety-seeking behavior. According to the elaboration likelihood model (Petty et al., 1983), consumers are more rational in consumption circumstances with a high level of situational involvement and tend to make decision based on their preference, thus less likely to avoid uncertainty using heuristic tactics such as variety seeking. In contrast, in consumption contexts of low-situational involvement, consumers tend to process information bypassing the peripheral route and thus are more likely to seek variety in products-related decision-making.

H3: Consumers’ involvement moderates the relationship between AI (vs. human) service environment and consumers’ variety-seeking behaviors, such that the relationship is stronger for low- than high-situational involvement.

## Overview of the Studies

**FIGURE 1:**
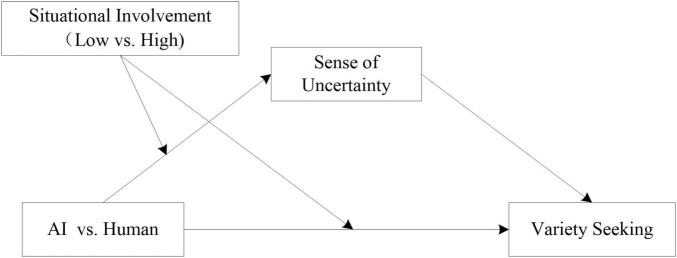
Conceptual model.

Variety seeking has a crucial influence on consumer decision-making. However, although individual characteristics and situational variables affect variety seeking, less is known about whether AI service context might impact the level of consumers’ variety seeking. Three studies demonstrate the consistent influence of AI service context on variety-seeking behaviors (Figure 1). Variety seeking will be higher when consumers are making decision in AI (vs. human) service environment (study 1). Furthermore, we investigated the proposed process through both mediation and moderation. As hypothesized, the impact of AI service context on variety seeking was mediated by a sense of uncertainty (study 2). The effects were moderated by situational involvement. Specifically, in contexts of high-situational involvement, the consumers are less likely to seek variety, and in contexts of low-situational involvement, they prefer more variety (study 3).

## Study 1: AI Service Context Increases Variety Seeking

Study 1 attempted to test our basic prediction of the AI service effect, i.e., variety seeking will be higher (vs. lower) when consumers are making decision in AI (vs. human) service context (H1).

### Methods

One hundred thirty-three undergraduate students (72 male; *M*_age_ = 21.86 years) in a major Asian university participated in this study for a payment of approximately US$0.40 each. The study used a between-subjects design with two (assistant type: AI vs. human) conditions.

We manipulated the assistant type by informing participants to imagine that when they enter a grocery, they are met with the following scene. In the AI (vs. human) condition, the participants were told that they could tell the service robot (vs. server) what they need by voice. To assess variety seeking, participants were given a shopping task and were asked to choose Kiss chocolates; they were exposed to two bags of food pictures, i.e., five different colored Kisses chocolates vs. the same-colored Kisses chocolates. After indicating their choices, participants answered a set of questions concerning to what degree they liked chocolates in their daily life and to what degree they were familiar with AI service (all scales: 1 = lowest, 7 = highest) and demographic questions. Finally, we asked participants whether they are currently losing weight or have a weight loss plan to rule out the alternative explanations and eliminated this part of the participants.

### Results

#### Variety Seeking

Participants’ choices were consistent with H1, such that variety seeking will be higher (vs. lower) when participant consumers are making decision in AI (vs. human) service environment. Participants in the AI condition were more likely to choose the five different colored Kisses chocolates over the same colored [62 vs. 38%, χ*^2^* (1) = 3.40, *p* < 0.05], whereas the difference in choice shares in the human condition was not significant. General liking chocolates [*M*_*AI*_ = 4.16, *SD* = 1.57 vs. *M*_human_ = 4.34, *SD* = 1.82, *F*(1, 131) = 0.36, *p* = 0.55], and familiarity with the AI service [*M*_*AI*_ = 4.25, *SD* = 1.44 vs. *M*_human_ = 4.45, *SD* = 1.40, *F*(1, 131) = 0.63, *p* = 0.43] did not vary between the service conditions.

### Discussion

Study 1 provides evidence that variety seeking will be higher (vs. lower) when the service is led with the intervention of an AI. Next, we examined uncertainty as the mechanism underlying this effect.

## Study 2: Sense of Uncertainty Mediates the Effect of Assistant Type

The focal objective of study 2 was to examine the mediating role of the sense of uncertainty and to replicate the effect of assistant type with another new context. In addition, we measured mood and ruled out such alternative explanation.

### Methods

A total of 107 undergraduate students (56 male; *M*_age_ = 20.70 years) in a major Asian university participated in this study for a payment of approximately US$0.40 each. The study used a between-subjects design with two (assistant type: AI vs. human) conditions.

We manipulated the assistant type by informing participants to imagine that they were doing their weekly grocery shopping at a nearby grocery store^1^. In the AI condition, the participants were told that they could tell the service robot what they need by voice, and no employee was involved. In contrast, in the human condition, the participants were told that they could tell the service staff what they need. Then, participants reported their sense of uncertainty by answering three questions: “I have a sense of uncertainty in the service,” “I think there is a lot of uncertainty about service interaction,” and “I am not sure the service can meet my expectation” (all on 7-point scales; α = 0.88) (Lee and Qiu, 2009). In addition, participants answered a set of questions concerning their mood with three positive emotions (i.e., happy, excited, and positive) and three negative emotions (i.e., sad, anxious, negative; all on 7-point scales). To assess variety seeking, we asked participants to choose seven yogurts from seven flavors (i.e., plain, strawberry, blueberry, vanilla, lemon, chocolate, and nuts). You can pick any seven flavors you want, and you can pick again as long as the total number of yogurts is seven. The number of unique yogurts represented a measure of variety seeking. Then, we asked participants whether they are currently losing weight or have a weight loss plan to rule out the alternative explanations, and eliminated this part of the participants. Finally, we asked them demographic questions, height, and weight.

### Results

#### Variety Seeking

To test our main prediction, we conducted a one-way ANOVA on variety seeking. As expected, participants in the AI condition chose more yogurt flavors than those in the human condition [*M*_AI_ = 4.22, *SD* = 1.90 vs. *M*_human_ = 3.37, *SD* = 1.91, *F*(1, 105) = 5.35, *p* < 0.05]. Participants in the AI condition displayed a greater variety-seeking tendency were similar to study 1, again supporting H1.

#### Sense of Uncertainty as the Mediator

**FIGURE 2:**
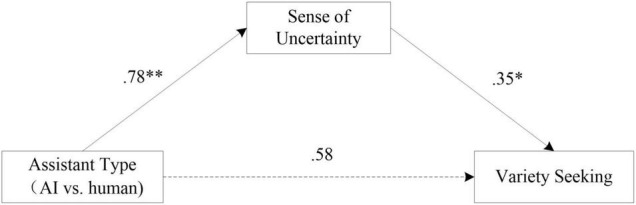
Mediation model in study 2. ^**^Significant at the 0.01 level; *significant at the 0.05 level.

A one-way ANOVA showed that compared with human condition, participants made their decision choice with the intervention of an AI that significantly increased their sense of uncertainty [*M*_AI_ = 4.65, *SD* = 1. 26 vs. *M*_human_ = 3.88, *SD* = 1.47, *F*(1, 105) = 8.67, *p* < 0.01]. Regression analyses further showed that AI condition significantly increased participants’ sense of uncertainty (β = 0.78, *t* = 2.94, *p* < 0.01), and sense of uncertainty significantly increased variety seeking (β = 0.35, *t* = 2.60, *p* < 0.05). We then conducted mediation analyses using Hayes’s PROCESS Model 4, and the bias-corrected bootstrap confidence intervals (CIs) were obtained for the contrast (AI vs. human conditions) using 5,000 bootstrap samples (Hayes, 2017). The results indicated that the sense of uncertainty mediated the relationship between service context conditions and variety seeking (indirect effect: β = 0.27, *SE* = 0.15, 95% CI = [0.06, 0.66]). The direct effect was not significant (β = 0.59, *SE* = 0.37, 95% CI = [−0.16, 1.32], see Figure 2), supporting H2.

#### Ruling Out Alternative Accounts

To rule out mood as an alternative explanation, we tested the effect of assistant type on the positive mood, such as happy [*F*(1, 105) = 0.05, *p* = 0.82], excited [*F*(1, 105) = 0.43, *p* = 0.51], and positive [*F*(1, 105) = 0.03, *p* = 0.87], and negative mood, such as sad [*F*(1, 105) = 0.44, *p* = 0.51], anxious [*F*(1, 105) = 0.28, *p* = 0.60], and negative [*F*(1, 105) = 0.12, *p* = 0.74], and the results showed no significant difference. This ruled out the possibility that the effect of assistant type conditions on variety seeking was due to mood. General liking yogurt [*M*_AI_ = 4.67, *SD* = 1.63 vs. *M*_human_ = 4.94, *SD* = 1.51, *F*(1, 105) = 0.78, *p* = 0.38], familiarity with the AI service [*M*_AI_ = 4.33, *SD* = 1.36 vs. *M*_human_ = 4.12, *SD* = 1.89, *F*(1, 105) = 0.45, *p* = 0.51], and BMI [*M*_AI_ = 20.77, *SD* = 4.15 vs. *M*_human_ = 20.48, *SD* = 3.33, *F*(1, 105) = 0.15, *p* = 0.70] did not vary between the AI and human conditions.

### Discussion

Study 2 provides further evidence for our predictions. The results show that participants are more willing to choose more flavors of yogurt when they are ordering food on AI than human condition (H1). More importantly, it confirmed that the sense of uncertainty is the underlying mechanism of the effect (H2). AI condition increased the sense of uncertainty, which led to a higher variety seeking. In addition, this study ruled out the alternative explanation based on mood. Next, we examined situational involvement as a moderator.

## Study 3: The Moderating Role of Situational Involvement

Thus far, we have shown the positive effect of AI condition on heightened sense of uncertainty and higher variety seeking. The objective of this final study is to identify a boundary condition of situational involvement and further validate the mediation effect of the sense of uncertainty.

### Methods

A total of 389 subjects (166 male; *M*_age_ = 28.33 years) in a major Asian university participated in this study for a payment of approximately US$0.40 each. The study used a 2 (assistant type: AI vs. human) × 2 (situational involvement: low vs. high) between-subjects design.

The manipulation of assistant type was similar to study 2; we asked participants to imagine that they were shopping at a nearby grocery store. Participants were presented with two choices of potato chips, each containing nine small packets (three different brands vs. nine different brands) that they want to purchase (see text footnote 1). In this study, we measured their relative preference for the two options (1 = definitely prefer three different brands, 7 = definitely prefer nine different brands), and the higher ratings implied higher variety seeking. We manipulated the situational involvement by means of the instructions given to participants before looking at the potato chips. In the low-situational involvement scenario, participants were told to imagine themselves buying snacks to eat at home alone. In the high-situational involvement scenario, participants were told to imagine themselves buying snacks to eat together for friends’ birthday celebrations. After indicating their preference, participants reported their sense of uncertainty (α = 0.94, all questions are the same as those in study 2), involvement (α = 0.82, 1 = unimportant, 7 = important; 1 = mean nothing to me, 7 = mean a lot to me; 1 = insignificant, 7 = significant; Zaichkowsky, 1985), and demographic questions.

### Results

#### Manipulation Check

A two-way ANOVA on situational involvement showed that perceived involvement was higher when participants eat together for friends’ birthday celebrations than eat at home alone [*M*_*low*_ = 3.85, *SD* = 1.88 vs. *M*_*high*_ = 5.05, *SD* = 1.51, *F*(1, 385) = 48.42, *p* < 0.001]. There was no main effect of assistant type condition [*F*(1, 385) = 0.20, *p* = 0.66] or interaction of involvement with assistant type on perceived involvement [*F*(1, 385) = 2.24, *p* = 0.14].

#### Variety Seeking

**FIGURE 3:**
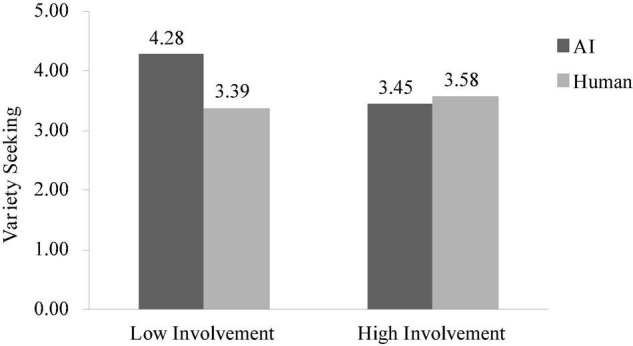
Interaction effect on variety seeking in study 3.

A two-way ANOVA on variety seeking revealed no significant main effect of assistant type conditions [*F*(1, 385) = 2.58, *p* = 0.11] or involvement conditions [*F*(1, 385) = 1.84, *p* = 0.18], but an anticipated significant interaction between these two factors [*F*(1, 385) = 4.61, *p* < 0.05]. Specifically, in the low involvement condition, participants prefer more variety when they interact with AI (vs. human), replicating the findings of studies 1 and 2 [*M*_AI_ = 4.28, *SD* = 2.34 vs. *M*_human_ = 3.39, *SD* = 2.29, *F*(1, 385) = 7.02, *p* < 0.01]. However, in the high involvement condition, the effect was attenuated [*M*_AI_ = 3.45, *SD* = 2.34 vs. *M*_human_ = 3.58, *SD* = 2.42, *F*(1, 385) = 0.15, *p* = 0.70, see Figure 3].

#### Sense of Uncertainty as the Mediator

**FIGURE 4:**
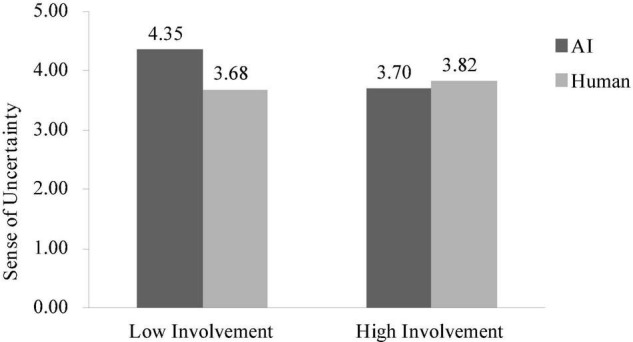
Interaction effect on the sense of uncertainty in study 3.

A two-way ANOVA on sense of uncertainty revealed no significant main effect of assistant type conditions [*F*(1, 385) = 2.66, *p* = 0.10] or involvement conditions [*F*(1, 385) = 2.22, *p* = 0.14], but an anticipated significant interaction between these two factors [*F*(1, 385) = 5.60, *p* < 0.05]. Specifically, in the low involvement condition, participants perceived higher uncertainty when they interact with AI (vs. human) [*M*_AI_ = 4.35, *SD* = 1.50 vs. *M*_human_ = 3.68, *SD* = 1.74, *F*(1, 385) = 7.96, *p* < 0.01]. However, in the high involvement condition, the effect was attenuated [*M*_AI_ = 3.70, *SD* = 1.68 vs. *M*_human_ = 3.82, *SD* = 1.70, *F*(1, 385) = 0.27, *p* = 0.60, see Figure 4].

To test the mediating effect of sense of uncertainty and the moderating effect of situational involvement, we ran a moderated mediation model applying the bootstrapping approach (Hayes, 2017, model 8), applying the 95% CI using 5,000 bootstrap samples. Results showed that bootstrapped confidence intervals suggested that the effect of assistant type conditions on sense of uncertainty was moderated by situational involvement (β = 0.35, *SE* = 0.16, 95% CI = [0.08, 0.72]). Specifically, in the low involvement condition, sense of uncertainty enhanced variety seeking (β = 0.29, *SE* = 0.12, 95% CI = [0.10, 0.57]), but in the high involvement condition, the path was not significant (β = −.05, *SE* = 0.10, 95% CI = [−0.28, 0.16]). The direct effects were not significant in either the low (β = −0.07, *SE* = 0.10, 95% CI = [−0.53, 0.40]) or the high involvement condition (β = 0.46, *SE* = 0.10, 95% CI = [−0.03, 0.38]).

### Discussion

We tested the moderating effect of situational involvement and further confirmed the sense of uncertainty as the underlying mechanism of the effect. Consistent with our prediction, consumers’ involvement moderates the relationship between AI (vs. human) service environment and consumers’ variety-seeking behaviors, such that the relationship is stronger for low- than high-involvement products. Especially, when the service is led with the intervention of an AI (vs. human) in the low-involvement condition, consumers will perceive higher uncertainty, which increases their variety seeking. However, in the high-involvement condition, assistant type does not affect consumers’ preferences.

## General Discussion

### Summary of Studies

Three studies demonstrate the consistent influence of AI service context on variety-seeking behaviors. Variety seeking will be higher when consumers are making decision in AI (vs. human) service environment. Furthermore, we tested the hypothesized mediating and moderating effects. As predicted, AI service context’s influence on variety seeking was mediated by the sense of uncertainty (study 2). The effects were moderated by situational involvement. Specifically, in consumption contexts of high-situational involvement, the consumers are less likely to seek variety, and in consumption contexts of low-situational involvement, they prefer more variety (study 3).

### Contributions and Future Research

These findings contribute in a number of ways. First, the findings shed light on drivers of variety seeking. While previous research has found several reasons for people’s need for variety (e.g., reducing satiation, McAlister, 1982; Sevilla et al., 2019; hedging against future uncertainty, Kahn and Lehmann, 1991; Ariely and Levav, 2000), the findings of this study demonstrate that AI service context encourages consumers to seek more variety in purchasing, which contribute to the antecedents of consumers variety-seeking behavior. Second, the results improve further understanding of artificial intelligence and service robots, which claim to boost efficiency and cut costs, resulting in a huge surge in the sales of service robot (Wirtz et al., 2018). Nevertheless, marketing research on AI service’s influence on consumer decision-making is scarce. Compared with service employees, service robots implicate various unique characteristics. For example, service robots do not demonstrate heterogeneity over time or between robots, and as a result, they are immune to human mistake and tiredness and adapt to the service context with great reliability (Huang and Rust, 2018). Apart from this, service robots will be unable to experience and express real emotions (Jerger and Wirtz, 2017). Due to these characteristics of service robots, the findings of this study help to explain why consumers would behave differently when purchasing in an AI service context compared with when purchasing in a typical human service setting.

Future work might examine other effects of AI service context. AI service has many downstream effects that may be of interest to marketing researchers. For example, AI service context might influence novelty seeking, *status quo* biases, impulsivity, or willingness to accept default settings. Consumers might be more willing to try new products when they are greeted by service robots, for example, and consumers might be less prone to purchase products or brands they are familiar with if they go shopping in supermarkets with AI service context rather than human service context. AI service context may also influence consumers’ affective reaction. For example, in AI service context, consumers may experience pleasant or curious reaction, which may also have a downstream effect on their behaviors.

### Managerial Contribution

The potential managerial implications of these findings are in the following aspects. To begin with, the effectiveness of variety appeals is likely to differ depending on the selling situation. For example, advertisements that appeal to variety seeking should be more effective in the context of AI services. As a result, products with inherently high (vs. low) variety may choose to focus their advertising by highlighting the AI characteristics of the context. Products, like yogurts, which naturally elicit variety, may be better selling with the help of an AI service robot. Likewise, what features to highlight in interacting with consumers may also differ. For example, when advertising with AI, a yogurt producer may want to focus on the variety of flavors consumers can choose from. Second, according to the findings, product offerings should be tailored based on selling contexts. For example, if AI robots are employed, supermarkets should highlight a variety in the categories of products.

## Data Availability Statement

The raw data supporting the conclusions of this article will be made available by the authors, without undue reservation.

## Ethics Statement

The studies involving human participants were reviewed and approved by the Tianjin University of Technology. The patients/participants provided their written informed consent to participate in this study.

## Author Contributions

All authors listed have made a substantial, direct, and intellectual contribution to the work and approved it for publication.

## Conflict of Interest

The authors declare that the research was conducted in the absence of any commercial or financial relationships that could be construed as a potential conflict of interest.

## Publisher’s Note

All claims expressed in this article are solely those of the authors and do not necessarily represent those of their affiliated organizations, or those of the publisher, the editors and the reviewers. Any product that may be evaluated in this article, or claim that may be made by its manufacturer, is not guaranteed or endorsed by the publisher.

## Footnotes

1 Visual examples available upon request from the corresponding authors.
